# Selected Biomarkers of Oxidative Stress and Energy Metabolism Disorders in Neurological Diseases

**DOI:** 10.1007/s12035-023-03329-4

**Published:** 2023-04-11

**Authors:** Izabela Korczowska-Łącka, Mikołaj Hurła, Natalia Banaszek, Dominik Kobylarek, Oliwia Szymanowicz, Wojciech Kozubski, Jolanta Dorszewska

**Affiliations:** 1 Laboratory of Neurobiology, Department of Neurology, Poznan University of Medical Sciences, 49, Przybyszewskiego St, 60-355 Poznan, Poland; 2 Chair and Department of Neurology, Poznan University of Medical Sciences, Poznan, Poland

**Keywords:** Biomarkers, Energy disturbances, Neurological diseases

## Abstract

Neurological diseases can be broadly divided according to causal factors into circulatory system disorders leading to ischemic stroke; degeneration of the nerve cells leading to neurodegenerative diseases, such as Alzheimer’s (AD) and Parkinson’s (PD) diseases, and immune system disorders; bioelectric activity (epileptic) problems; and genetically determined conditions as well as viral and bacterial infections developing inflammation. Regardless of the cause of neurological diseases, they are usually accompanied by disturbances of the central energy in a completely unexplained mechanism. The brain makes up only 2% of the human body’s weight; however, while working, it uses as much as 20% of the energy obtained by the body. The energy requirements of the brain are very high, and regulatory mechanisms in the brain operate to ensure adequate neuronal activity. Therefore, an understanding of neuroenergetics is rapidly evolving from a “neurocentric” view to a more integrated picture involving cooperativity between structural and molecular factors in the central nervous system. This article reviewed selected molecular biomarkers of oxidative stress and energy metabolism disorders such as homocysteine, DNA damage such as 8-oxo2dG, genetic variants, and antioxidants such as glutathione in selected neurological diseases including ischemic stroke, AD, PD, and epilepsy. This review summarizes our and others’ recent research on oxidative stress in neurological disorders. In the future, the diagnosis and treatment of neurological diseases may be substantially improved by identifying specific early markers of metabolic and energy disorders.

## Introduction

**Fig. 1:**
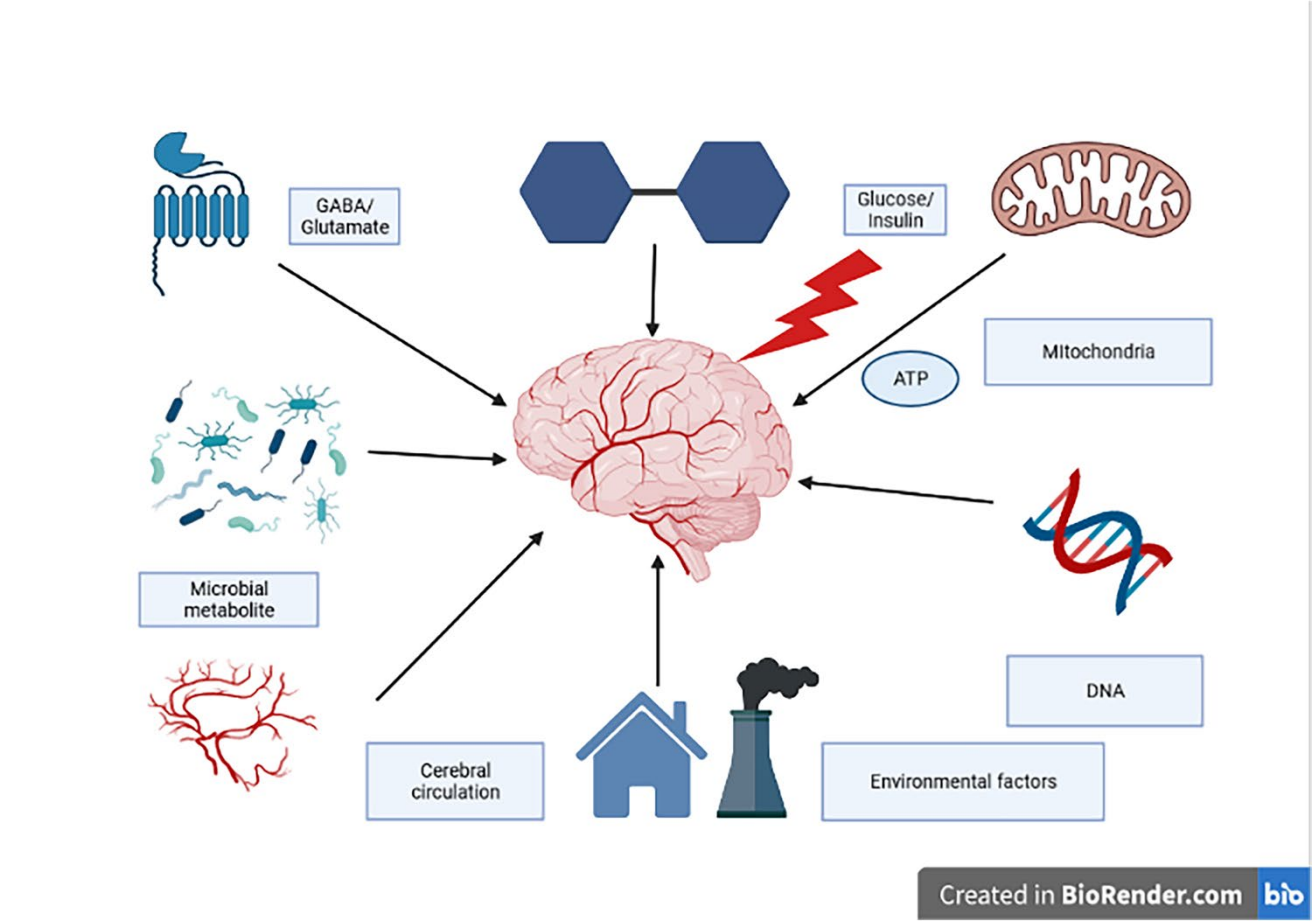
The energy request of the brain under physiological and pathological conditions. ATP, adenosine triphosphate; GABA, gamma-aminobutyric acid

The brain makes up 2% of the average person’s weight. Nevertheless, while working, it uses as much as 20% of the energy obtained by the body [1]. The brain is responsible for a large proportion of the brain’s oxygen consumption [2, 3]. It is estimated that neural signaling accounts for about 75% of cortical energy consumption, while the remaining 25% is used to maintain other functions. Out of the 75%, 44% is used for synaptic transmission and 16% for action potentials [3]. The source of energy for the work of the brain is mostly exclusively glucose [4]; Lundgaard et al. [5] have shown that neurons, and not astrocytes, during rest as well as neural activity, are the primary consumers of glucose (Fig. 1, Table 1).

**Table 1:** Biomarkers of energy disorders in neurology

Biomarkers	Stroke	Alzheimer’s disease (AD)	Parkinson’s disease (PD)	Epilepsy
Glucose - Glucose metabolism - Lactate - Glycogen - Insulin - 2-Deoxyglucose - Astrocyte activity	- Glycemic disorders - Reduction of lactate production and monocarboxylic acid transporter expression - Glycogen synthase kinase-3 activation - Reduction of ischemic brain damage by 2-deoxyglucose - Insulin and insulin-like growth factor 1 (IGF-1) - Neuroprotective factors Ref. [170–174]	- Disorders of cerebral glucose metabolism - Glucose transportation abnormality - Intracellular glucose catabolism dysfunction - Cerebral glucose hypometabolism - Astrocyte reactivity—an early feature of AD - Insulin signaling dysfunction - 18 F-Fluoro-2-deoxyglucose positron emission tomography- cognitive impairment biomarker Ref. [175–179]	- Glucose metabolism - dysregulation - Impaired glucose control - Glycogen synthase kinase-3beta (GSK-3beta) presence in Lewy bodies (LBs) - Impaired adaptive insulin response - 18 F-Fluoro-2-deoxyglucose positron emission tomography - imaging biomarker for idiopathic PD and atypical parkinsonism associated with dementia - Astrocytes activity - Key regulators of inflammatory responses Ref. [180–183]	- Impairments in brain glucose metabolism - Increase in lactate levels - Increase the blood level of insulin - 18 F-Fluoro-2-deoxyglucose—a decrease of glucose metabolism in the interictal functional deficit zone - Impairment of astrocyte function and energy homeostasis Ref. [25, 156, 184–187]
Mitochondria - Dysfunction - Reactive oxygen species (ROS) - Electron transport chain	- Neural cell death during ischemic stroke - ROS generation, apoptosis, and electron transport chain dysfunction Ref. [188]	- Mitochondrial dysfunction - Disruption in nuclear-encoded oxidative phosphorylation subunits (OXPHOS) - Severely disturbed mitochondrial activity, especially complex IV - ROS generation - Cytochrome c oxidase (CO) dysregulation Ref. [12, 111–117, 175]	- Mitochondrial dysfunction - Severely disturbed mitochondrial activity, especially complex I and III - ROS generation - Mitophagy Ref. [12, 131, 132]	- Mitochondrial dysfunction - ROS generation - Change in energy level - Alteration in the signaling cascade of apoptosis Ref. [158, 189, 190]
DNA - Oxidation - Damage	- DNA lesions - Defective DNA repair Ref. [191]	- Damage to both DNA (mitochondrial, mtDNA, and nuclear, nDNA) - Aggregation of advanced glycation products (AGE) - 8-Oxo-2-deoxyguanine (8-oxo2dG) generation - DNA oxidative modification - Impaired DNA repair processes Ref. [10, 12–15, 20, 21, 101, 104, 105]	- Nucleic acids damage - 8-Oxo2dG generation - DNA alterations Ref. [15, 16, 147, 148]	- DNA damage - Accumulation of more DNA damage foci and more susceptibility to cell death Ref. [169, 192]
Biothiols - Homocysteine (Hcy) - Cysteine (Cys) - Methionine (Met) - Glutathione (GSH) - Vitamin B6 - Vitamin B12 - Asymmetric dimethylarginine (ADMA)	- Hcy increases stroke risk - Hcy activates the apoptosis program in nerve cells - Induces neuronal metabolic dysfunction - Aggravates atherosclerosis - Has a toxic effect on the endothelium of blood vessels - Increases changes in vascular smooth muscle - Provokes the inflammatory process - Causes oxidative stress and enhances the production of fibrinogen - ADMA damages endothelial cells - GSH—no correlation with stroke Ref. [55–59, 61, 62, 82, 87, 88]	- Hcy increases AD risk - Correlates with AD severity - Leads to the development of dementia - Inhibits GSH production - Oxidative stress generations - Alters thiamine metabolism - GSH’s potential role as a predictive biomarker for AD development - ADMA interacts with Hcy metabolism and may contribute to neurodegeneration and accumulation of phosphorylated tau in AD Ref. [15, 26, 107–110, 175, 193]	- Hcy may be associated with the development of PD - Hcy induces nerve cell apoptosis and oxidative stress, and DNA damage - GSH contributes to the development of PD - High ADMA and NO levels in patients with PD lead to endothelial dysfunction Ref. [15, 194–196]	- Hcy risk factor for vascular diseases - Induces apoptosis - Toxic effects on both the vascular and nervous systems - ADMA regulates the level of Hcy - Disturbed GSH level Ref. [167, 168, 197]
Genetic variants - Genetic variants - Epigenetics	- Variants of genes involved in Hcy metabolism and repair of DNA damage - Epigenetic regulation of histone modifications, DNA methylation, and activity of non-coding RNAs Ref. [75, 80, 191, 198]	- Variants of genes involved in Hcy metabolism and repair of DNA damage - Genes associated with ROS generation - Epigenetic regulation of histone modifications, DNA methylation, and activity of non-coding RNAs Ref. [14, 15, 113, 117, 199]	- Variants of genes involved in Hcy metabolism and repair of DNA damage - Genes associated with ROS, and oxidative stress generation - Epigenetic regulation of DNA methylation, histone modifications, and altered microRNA expression Ref. [14, 15, 131–138, 193, 200]	- Variants of genes involved in Hcy metabolism and repair of DNA damage - Genes associated with oxidative stress generation - Epigenetic regulation of DNA methylation, particularly hypermethylation - Posttranscriptional modification of histones and non-coding RNAs Ref. [167–169, 201]
Neurotransmitters - Glutamate - Gamma-aminobutyric acid (GABA)	- Increase extracellular glutamate - Glutamate contributes to brain damage in ischemia Ref. [202]	- Glutamate and GABA provide a balance between excitation and inhibition - Provide glutamatergic, GABAergic signaling in microglia-neuronal cross-talk in AD - Important for glutamate/GABA-mediated dialogue between microglia and neurons Ref. [203]	- Increase glutamate level - Excitotoxicity induced by glutamate - Impairment of the ability of glial cells to reuptake and respond to glutamate - Development of nerve cell damage Ref. [204]	- A disorder of neurotransmitters levels, especially GABA and glutamate - Dysfunctional glutamate metabolism Ref. [157–159]
Pharmacotherapy - L-Dopa - Antiepileptic drugs (AEDs)	No literature data	- AED improvement of cognitive functions - Neuroprotective efficacy of AEDs - Disturbs the metabolism of biothiols Ref. [205]	- AED improvement of cognitive functions, essential tremor, spasticity - L-Dopa leads to an increase in oxidative stress and DNA damage - Generates 8-oxo2dG - Disturbs the metabolism of biothiols Ref. [148, 206]	- AED disturbs the metabolism of biothiols - Introduction of cells to apoptosis Ref. [167–169]

Mitochondria are essential sources of energy for the brain [6]. These organelles convert energy to adenosine triphosphate (ATP), which neurons need to regulate, among other neurotransmissions [1]. Moreover, ATP plays a crucial role in processes related to maintaining neuronal plasticity [7, 8]. Mitochondria are not only the main energy producers in cells but also the main source of reactive oxygen species (ROS). They generate ROS in oxidative phosphorylation, which is also their major source. ROS leads to damage to macromolecular compounds and metabolic disturbance. Free lipid radicals (lipid peroxides) are formed during lipid oxidation and trigger a destructive chain reaction. They are included in the body due to metabolic reactions, especially in burning polyunsaturated fatty acids, leading to damage to the cell membrane. As a result of damage to the cell membrane, the cell cannot perform its proper metabolic functions, leading to the development of functional disorders in the brain and other organs [9–11].

Excitotoxicity and disrupted energy metabolism are significant events leading to nerve cell death in neurodegenerative disorders. These cooperative pathways share one common aspect: triggering oxidative stress by ROS formation. The following oxidative stress markers (8-oxo-2-deoxyguanine, 8-oxo2dG; homocysteine, Hcy; sirtuin 1, SIRT1; short-chain fatty acids, SCFA; others described in the review) could be of interest while studying central nervous system (CNS) diseases.

Oxidative damage is assessed by analyzing lipid peroxidation, mitochondrial dysfunction, and DNA damage. The biomarkers of DNA oxidative damage include 8-oxo2dG [12–15]. 8-Oxo2dG is the product of the oxidation of guanine in DNA. This oxidative change results in G-C transversion to T-A [16–18]. The presence of 8-oxo2dG in nucleic acid during replication may lead to somatic and gene function loss in up to 14% of events, followed by the production of non-functional proteins, including abnormal DNA repair enzymes [19].

Elevated levels of 8-oxo2dG have been demonstrated in the brain and lymphocytes of Alzheimer’s disease (AD) [13, 14] and Parkinson’s disease (PD) [12] patients. At the same time, it has been shown that an increased level of this marker may indicate a gradual increase in damage to nucleic acids and the development of neurodegeneration. Studies by Dorszewska et al. [15] and Dezor et al. [13] have shown that the elevated level of 8-oxo2dG indicates not only the severity of AD dementia (on the Mini–Mental State Examination; MMSE scale) but also the effectiveness of DNA damage repair. Moreover, Dorszewska et al. [14] showed that the repair process of 8-oxo2dG involves three OGG1 isoforms, 1a, 1b, and 1c, and that OGG1-1c most likely plays the role of a compensatory system in the initial phase of the ongoing degenerative process in AD. On the other hand, the polymorphism of the OGG1 Ser326Cys gene is related to the pathogenesis of this dementia through the control of DNA oxidative modification.

Another biomarker involved in regulating mitochondrial functions, stabilizing the chromatin structure, and promoting the DNA repair process, is SIRT1. It is a deacetylase responsible for mitochondrial biogenesis, glucose metabolism, inflammation, and gene transcription. SIRT1 is encoded by the *SIRT1* gene, whose polymorphisms are related to physiological aging and neurodegenerative diseases. The role of the rs7895833 polymorphism in increased life expectancy has also been proposed since the AG genotype is associated with higher levels of SIRT1 and lower body weight obesity index [20, 21].

Microbiota is also involved in energy regulation in the brain; it suggests that microbial metabolites influence its functioning. These include SCFA with butyric acid. Butyrate is considered the regulator of microbe levels in the body and is involved in energy metabolism and the control of immune function. Butyrate affects multiple host physiological processes via specific transporters and receptors and as a histone deacetylases (HDAC) inhibitor. It promotes histone acetylation and stimulation of gene expression. Receptors such as GPR43/FFAR2, GPR41/FFAR3, and GPR109a/HCAR2 and transporters including MCT1/SLC16A1 and SMCT1/SLC5A8 are important for its operations [22, 23].

Butyric acid may be involved with other SCFAs such as acetate, propionate, acetoacetate, and d-β-hydroxybutyrate in the pathogenesis of many diseases, including those of a neurological type (e.g., AD) and diabetes, arrhythmogenic cardiomyopathy. Butyrate as an experimental drug has been used in experimental models of neurological disorders, from depression to neurodegenerative diseases of old age [23].

Disruption of bioenergetics may correlate with physiological aging [9–11] and age-related diseases, including AD (see the section entitled “Energy Changes in Alzheimer’s Disease”), PD (see the section entitled “Energy Disorders in Parkinson’s Disease”), stroke, and epilepsy [12, 24, 25].

Moreover, the interaction between oxidative stress and neuro-inflammation leads to β-amyloid (Aβ) generation in the brain of AD patients, while PD is the accumulation of degradation of dopaminergic neurons in the *substantia nigra*, due to the greater susceptibility of the nigrostriatal circuit to mitochondrial dysfunction and oxidative stress. Additionally, mitochondrial complex I activity, which is reduced in PD and is critical to dopaminergic neuron survival, is compromised when glutathione (GSH) levels are reduced in combination with the generation of peroxynitrite radicals. Administration of levodopa (L-dopa) to PD patients, especially long-time therapy, may cause side effects in the form of increased neurotoxicity. The augmented oxidative stress in patients treated with L-dopa may result from lowered levels of GSH and excessive oxidation of dopamine (DA) [12].

GSH in mitochondria (MtGSH) is an alternative pool for cytoplasmic GSH, and its disturbance may lead to the overproduction of ROS, mitochondrial failure, release of caspases, inducers of apoptosis, and the development of dementia. Moreover, during several neurodegenerative diseases, including AD, the level of GSH decreases. GSH has been proposed as a biomarker for diagnosing neurodegenerative diseases and a promising target for future therapies.

Another biothiol involved in the pathogenesis of AD and PD is excessive levels of Hcy [15, 26]. Hcy, a sulfur amino acid, is an intermediate metabolite of methionine. Elevated plasma Hcy level (hyperhomocysteinemia) may cause atherosclerotic vascular and neurodegenerative diseases such as AD and PD. Mild hyperhomocysteinemia is quite prevalent in the general population. It can be caused by genetic defects in the enzymes involved in Hcy metabolism, nutritional deficiencies in vitamin cofactors, certain medications, or renal disease. In the body, Hcy undergoes transsulfuration and transmethylation. Under physiological conditions, about 50% of Hcy is catabolized by transsulfuration to cysteine (Cys), and the remaining 50% of this biomarker is methylated to methionine (Met) [15] (see the chapter entitled “Stroke and energy disorders”).

Another neurological disorder in which biothiols are disturbed is ischemic stroke. In the brain, ischemic stroke is caused by the blockage or rupture of blood vessels. It leads to a disturbance of cerebral blood flow and reduced energy supply to neurons and other brain cells, ultimately resulting in the loss of neurons [27]. Other markers important for the pathogenesis of ischemic stroke are changes in the level of neurotransmitters stimulating, e.g., glutamate and aspartate, and the accompanying release of calcium ions and generation of oxidative stress [28]. Oxidative stress after experimental ischemia violates the integrity of the genome, causes DNA damage and death of neurons and glial and vascular cells, and may lead to the development of neurodegenerative diseases [29, 30]. Moreover, the effects of ischemic changes may have a long-term impact on central structures involving inflammatory factors and be associated with the formation of amyloid plaques and Alzheimer’s-type dementia [31, 32]. More information indicates that disorders in the microbiota-gut-brain axis, manifesting with age and the onset of ischemic stroke, may be associated with the development of risk factors for stroke [33]. Approximately 3% of patients with stroke develop epilepsy [34].

Seizures are the result of a sudden and temporary synchronization of neural activity. It is believed that astrocytes are involved in neurotransmitter storage as well as neurotransmission activity and are responsible for providing neurons with a high energy level to maintain regular and pathological activity, e.g., during an epileptic episode. Moreover, glial cells, especially astrocytes, are involved in neurotransmission through their effects on the transmission of neurons and synapses. The cause of the seizures results from an imbalance between the activity of excitatory and inhibitory neurons, especially between the glutamatergic and gamma-aminobutyric acid (GABAergic) systems. The imbalance between them may be the cause of abnormal bioelectric brain activity. Restoring the balance between excitatory and inhibitory neurons, including relevant pharmacotherapy, may lead to correct neurotransmission in the brain [25].

Emerging energy disorders in neurological diseases, due to the structural and functional complexity of the nervous system, make it necessary to monitor external and internal factors such as energy failure in mitochondria and cytosol, antioxidant status, and response to DNA damage [35, 36].

Energy disturbances in the brain can also be related to genetic or environmental factors such as stress, inappropriate diet, and exposure to toxins [4, 37].

Understanding the brain’s precise and complex energy mechanisms is crucial to discriminate between physiological and pathological processes and will allow indicating more precise disease biomarkers of neurological diseases.

## Methodology

The articles selected for the following review have been chosen based on multiple searches through public databases (PubMed, Scopus, Google Scholar, Embase) with the following criteria: “oxidative stress” + “molecular factors” + “ischemic stroke” (242 articles found and 49 results at Embase) and “oxidative stress” + “molecular factors” + “Alzheimer’s” (1134 articles found and 30 results at Embase) and “oxidative stress” + “molecular factors” + “Parkinson’s” (827 articles found and 28 results at Embase) and “oxidative stress” + “molecular factors” + “epilepsy” (101 articles found and 15 results at Embase). Additional papers have been selected based on a search through the references of eligible articles. The last search was performed in August 2022.

The authors’ own research on the subject was also used.

## The Role of Homocysteine and Its Metabolism in Stroke

According to the World Health Organization, stroke is the “incoming epidemic of the twenty-first century” [38]. In the past decade, the definition of stroke has been revised and is now defined as an episode of acute neurological dysfunction presumed to be caused by ischemia or hemorrhage, persisting for more than 24 h or leading to earlier death with no apparent cause other than vascular origin [39]. Stroke is the second leading cause of death and disability worldwide, accounting 2017 for 11% of women and 10% of men, while metabolic syndrome (Met/Syn) increases the risk of ischemic stroke [40]. Reaven and co-workers [41] systematically described and defined Met/Syn, which included hyperglycemia, abdominal obesity, hypertriglyceridemia, low high-density lipoprotein cholesterol concentration, hypertension, and interactions between genetic factors, sedentary lifestyle, and diet. Met/Syn is a result of risk factors of metabolic origin that are accompanied by increased risk for cardiovascular disease (consisting of atherogenic dyslipidemia, a prothrombotic state, a pro-inflammatory state, elevated blood pressure, and high-fasting glucose levels of either pre-diabetes or diabetes [42–44], obesity, and insulin resistance).

**Fig. 2:**
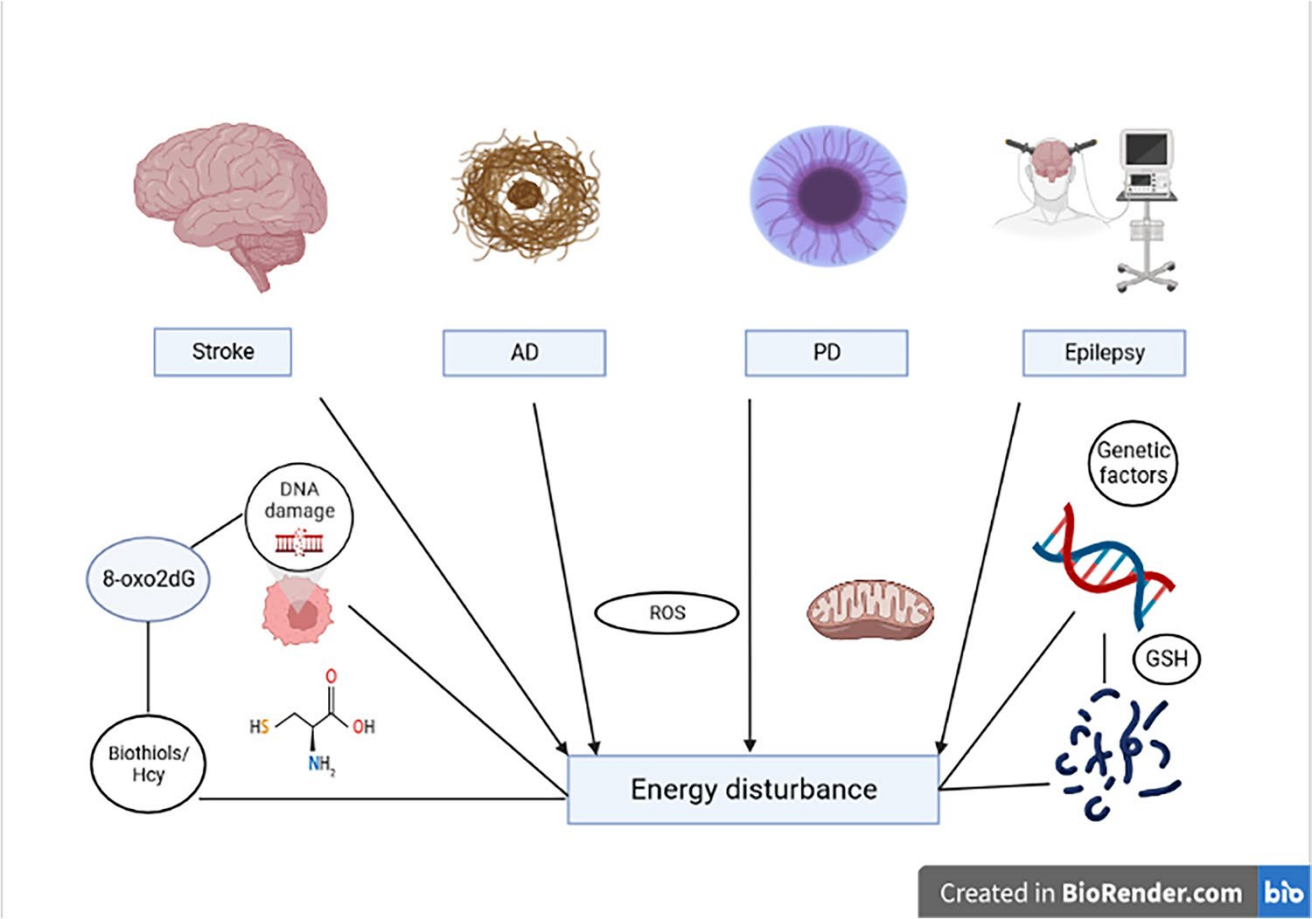
Biological markers related to oxidative stress in neurological diseases. AD, Alzheimer’s disease; PD, Parkinson’s disease; 8-oxo2dG, 8-oxo-2-deoxyguanine; Hcy, homocysteine; ROS, reactive oxygen species; GSH, glutathione

Studies into the traditional risk factors such as metabolic syndrome, smoking, and diets have found that Hcy is an independent risk factor for cerebrovascular diseases (Fig. 2). Hcy is a sulfhydryl-containing amino acid and is an important intermediate product of methionine and Cys metabolism. The concentration of Hcy blood (reference values 5–15 mol/l) should not exceed 14 mol/l on an empty stomach and 30 to 38 mol/l after 6 h from methionine loading. Genetic and nutritional factors, estrogen levels, and age affect the Hcy plasma level [45–47]. Subsequently, the predominant metabolism of Hcy occurs via three pathways as a remethylation cycle back to Met by methionine synthase (MS). Tetrahydrofolate metabolism provides a methyl group, and Hcy is remethylated to Met with the assistance of vitamin B12 (VitB12) [48] under the action of methionine adenosyltransferase (MAT). Met is metabolized to S-adenosine methionine (SAM), and SAM is one of the major methyl donors that can be converted to S-adenosine homocysteine (SAH), a methyl removed in this process, which is involved in epigenetic modifications under the action of methyltransferase [49]. SAH removes adenosine by S-adenosine homocysteine hydrolase (SAHH) to form Hcy [50], and these substances are well-associated with the Met cycle and energy metabolism. The second one is known as the transsulfuration pathway or transsulfuration to Cys with vitamin B6 (VitB6) as a coenzyme, whereby Hcy and serine are condensed into cystathionine under the catalysis of cystathionine β-synthase (CBS), followed by cystathionine catalyzed by γ-cystathionine lyase to produce Cys, that is oxidized to sulfate after a series of enzyme catalyzes, and excreted through the urine in the form of inorganic salts [47]. The third one is immediate release into the extracellular fluid; excessive Hcy is thought to be released from the intracellular to the extracellular fluid through the difference in internal and external concentrations and then exported to the systemic circulation to prevent its intracellular accumulation [48, 51]. VitB6, VitB12, and folic acid are involved in the metabolic pathways of Hcy in the methylation and transsulfuration cycle, the lack of which leads to the production of Hcy. Hence, the production and metabolic balance of Hcy are essential for maintaining the body’s homeostasis. Hcy causes platelet adhesion [52] and aggregation, injures tissue endothelial cells, and affects thrombin regulatory protein activity by inhibiting the binding of endothelial cells and tissue-type plasminogen activator [53, 54]. Studies suggest that Hcy induces hypertension by promoting toll-like receptor 4 (TLR-4-driven) chronic vascular inflammation and mitochondria-mediated cell death [55]. Hcy can activate the apoptosis program in nerve cells, inhibit cell membrane sodium/potassium enzyme, induce neuronal metabolic dysfunction [56, 57], and promote the occurrence of acute ischemic stroke [58]. Hcy aggravates atherosclerosis with elevated oxidative stress and reduces S-nitrosylation levels of redox-sensitive protein residues in the vasculature [59]. High levels of Hcy in the blood have a toxic effect on the endothelium of blood vessels and are associated with an increased risk of neurodegenerative processes [60]. Hyperhomocysteinemia activates the enzyme asymmetric dimethylarginine (ADMA). ADMA is an inhibitor of the endothelial isoform of nitric oxide synthase (eNOS), and a reduction in eNOS activity results in a decrease in nitric oxide (NO) concentration (decreasing NO concentration leads to disturbance of the balance between vasodilating abilities in favor of vasoconstriction capacity), which impairs the vasodilating capacity of vascular endothelial cells [61–63]. Hyperhomocysteinemia leads to impaired hemostasis resulting in the formation of blood clots in the blood vessel walls [64]. Methylenetetrahydrofolate reductase (MTHFR), CBS, and MS are the key enzymes in Hcy metabolism. MTHFR is an enzyme responsible for the remethylation of Hcy to Met, lowering the Hcy concentration. Studies have revealed that polymorphisms affecting the activity of this enzyme may occur within the *MTHFR* gene [65, 66]. Missense mutations change the amino acid structure of MTHFR and cause the enzyme N5-N10-methyltetrahydrofolate to be formed, characterized by reduced activity that promotes the development of hyperhomocysteinemia [67–69]. Hcy cannot be converted into methionine normally, causing a significant increase in the Hcy content in the blood, which—on the other hand—increases stroke susceptibility [70]. Studies reveal that the heterozygous mutation frequency of *MTHFR* C677T is higher than that reported in the Chinese population [71, 72], while the homozygous mutation frequency of *MTHFR* C677T in the Chinese population [73, 74] is the same as reported in non-Chinese. In Caucasian and African populations, Kumar et al. [75] conducted a meta-analysis that indicated that genotyping of the *MTHFR* gene A1298C polymorphism may be used as a predictor for the occurrence of ischemic stroke; it also resolved the correlation between *MTHFR* A1298C polymorphism and stroke susceptibility. It was discovered that *MTHFR* A1298C polymorphism was correlated with stroke in adults. However, the correlation between *MTHFR* A1298C polymorphism and stroke in children lacked corresponding evidence [76]. A study by Dong et al. [77] indicated that the *MTHFR* gene could encode the MTHFR enzyme, which plays a crucial role in regulating cellular Hcy and folate metabolism by catalyzing the conversion of 5,10-methylpentylenetetrahydrofolate to 5-methyltetrahydrofolate, and an elevated Hcy level in blood circulation is considered an independent risk factor for cerebral, coronary, and peripheral atherosclerosis [78]. The mutation frequency of *CBS* 844ins68 and *MS* A2756G plays an important role in the synthesis and metabolism of Hcy; in Chinese people, the mutation frequency of *MS* A2756G in the Chinese population was significantly lower than that in Caucasians [71]. A domestic meta-analysis [79] provided evidence that *CBS* T833C genetic polymorphism was associated with the risk of stroke. Nevertheless, the results from subgroups of the Chinese and Caucasian populations are different; in the Chinese subgroup, the results showed that *CBS* T833C polymorphism leads to increased incidences of stroke. The findings of several other studies suggest that a 3 mmol/L lower total Hcy level could be associated with a 10% lower risk of recurrent strokes [80–82]. Accordingly, clarifying the correlation between Hcy and ischemic stroke and reducing Hcy levels in at-risk patients may play a role in preventing ischemia. Many studies have shown that Hcy is closely correlated with ischemic stroke, especially in the small-vessel occlusion (SVO) and large-artery atherosclerosis (LAA) subtypes [83, 84]. However, some studies have indicated that Hcy is only related to LAA [85].

The precise mechanism of Hcy on the susceptibility of ischemic stroke remains unclear. Most studies of high Hcy levels as a cause of ischemic stroke have found that Hcy generates oxidative stress, damages endothelial cells, and increases fibrinogen production [86, 87]. Moreover, oxidative stress plays an essential role in acute ischemic stroke pathogenesis. Ozkul et al. [88] measured serum levels of NO, malondialdehyde (MDA), and GSH within the first 48 h of stroke in 70 patients and found them significantly higher in acute stroke patients. On the other hand, they did not find a statistically significant correlation between GSH levels and the malfunction of the CNS.

There is possibly a deleterious effect of oxidative stress on clinical outcomes in acute ischemic stroke, and the elevation of GSH levels may be an adaptive mechanism during these occurrences.

## Energy Changes in Alzheimer's Disease

The elongation of human survival accompanied significant progress in medicine and technique in the second half of the twentieth century. The elderly population in most modern economics implicates an array of factors contributing to age-related diseases, the most notable of which are epigenetic changes, inflammation, telomere shortening, and mitochondrial abnormalities [89–91]. Therefore, the problem of neurodegenerative disorders is becoming considerably more pressing. One of the most common neurodegenerative disorders is AD, the main cause of the severe decline of cognitive functions. Approximately once every minute, a US citizen is diagnosed with AD [92]. Statistical analysis shows that we might see substantial growth in diagnosed AD patients in the upcoming years, reaching as many as 1 million patients yearly [93–96]. Moreover, AD-induced cognitive impairment results in a significant decrease in quality of life [97].

Some of the most notable pathological changes ongoing in AD are Aβ plaques and hyperphosphorylated tau protein neurofibrillary tangles [98]. In AD development, oxidative stress plays a substantial role (Fig. 2). Researchers currently perceive protein oxidation, DNA oxidation, and lipid peroxidation as signs of energy disorders taking part in AD pathology [99]. ROS results from the O_2_ transformation intracellular that occurs in every human body cell [100]. Additionally, a certain balance between ROS production and elimination can be severely disrupted in energy disorders. ROS overproduction in such diseases is more likely to occur, leading to cell metabolism destabilization and oxidative damage to DNA and organelles [101]. Therefore, ROS levels could be one of the potential biomarkers of energy disorders in AD and Perez et al. [102] study showed that it is possible to assess the higher level of ROS in fibroblast samples taken from AD patients. The same cells may be the source of another biomarker, 8-oxoguanine. Its presence could indicate oxidative DNA damage in AD patients [102, 103]. Energy disorders might also stimulate the aggregation of advanced glycation products (AGE). Recent research has pointed to AGE as one of the factors playing a role in AD pathology [104]. Moreover, AGE has been identified in Aβ plaques; thus, AGE might be a future biomarker of AD; however, further research into the pathophysiological influence of AGE on AD is needed [104, 105].

Another emerging biomarker is Hcy. Numerous pathological processes occur in the hyperhomocysteinemic state, especially in the central nervous system. Furthermore, Hcy induces ROS production, mitochondrial imbalance, and lipid peroxidation, contributing to energy disorders [106]. Elevated Hcy concentration in the brain stimulates neurotoxicity and promotes cognitive decline [107]. Moreover, an elevated Hcy level has been associated with increased AD risk and can be correlated with the disease’s severity [107, 108]. All these points favor Hcy being an important AD biomarker; nevertheless, more research is needed [107–110].

Energy disorders contributing to AD might also originate from disruption in nuclear-encoded oxidative phosphorylation subunits (OXPHOS); as a result, mitochondrial activity is disturbed severely; especially, complex IV seems to be the most affected. This results in a chain of mitochondrial reactions ending in increased production of ROS. Lunnon et al.’s [113] study demonstrated that specific genes are more likely to invoke such a reaction, namely *MT-ND1*, *MT-ND2*, *MT-ATP6*, *MT-CO1*, *MT-CO2*, *MT-CO3*, and *MT-ND6*. Determining the overexpression of said *OXPHOS* genes might give some insight into energy disorder-driven AD pathology. Furthermore, *OXPHOS* genes may be a viable option for an AD biomarker in the future [111–116].

Another representative of mitochondria-oriented pathology is cytochrome c oxidase (CO) dysregulation. Among AD patients, the activity of CO is severely impaired [117]. In addition, impaired CO functionality might increase ROS production, leading to a higher chance of oxidative damage. Determining the specific mutations in patients might mean these changes can be another biomarker of energy disorders in AD. Therefore, the energetic disturbance in AD caused by pathological changes in the course of the disease may be the starting point for new therapies in the future.

## Energy Disorders in Parkinson’s Disease

PD is the second most prevalent neurodegenerative disorder after AD. Approximately 2% of the population over the age of 65 suffers from PD. Moreover, men are affected 1.5 times more than women [118]. The main symptoms of PD are resting tremor, rigidity, bradykinesia, and postural instability; however, the precise etiology is yet to be defined, although it should be considered a multifactored disease with environmental, genetic, and epigenetic risks. With increasing age, the risk of PD is greater. Pathologically, the disease is characterized by the loss of dopaminergic neurons in the *substantia nigra*, which leads to decreased DA levels in the basal ganglia and the formation of Lewy bodies (LB). These intracellular formations consist of α-synuclein conglomerates [119]. It is believed that the occurrence of motor symptoms is correlated with dysregulation of the basal ganglia circuitries [120]. Additionally, during PD, there is a tendency toward non-motor symptoms such as sleep disturbances, depression, cognitive deficits, and autonomic and sensory dysfunction [121]. Mostly, PD is sporadic and, as a cause of the occurrence, indicates a correlation between genetic and environmental factors. In 15% of cases, the cause of PD is rare familial genetic mutations. In both forms of PD, mitochondrial dysfunction has been observed [122].

The first case of PD was described over 200 years ago, but even currently, there are no specific diagnostic markers [123]. To recognize PD, the patient must represent the clinical symptoms, including motor and non-motor disorders [124]. Oxidative stress is indicated as one of the main factors responsible for neurodegeneration in PD [125]. ROS are produced in the mitochondrial respiratory chain (Fig. 2). They result from metabolic processes occurring in cells and play an important role in cell defense [126, 127]. The antioxidative mechanisms control the levels of ROS in dopaminergic neurons by including GSH, superoxide dismutase (SOD), and protein deglycase 1 (DJ-1). In patients with PD, more mutations have been described [125]. This statement may confirm that the nigral neurons are harmed by oxidative damage [128]. During aerobic respiration, electrons may escape from complex I (nicotinamide adenine dinucleotide, NADH) and complex III (cytochrome bc1) to generate ROS [129]. This resulting ROS may be converted further into hydrogen peroxide by SOD2 and detoxified by catalase [130]. If these mechanisms are inefficient, the reactive molecules oxidize mitochondrial DNA (mtDNA) [12]. Neuronal damage leading to the presence and progression of PD may be the result of these neuronal abnormalities [128].

Mitochondria need a constant rejuvenation. Maintaining a healthy mitochondrial population requires the clearance of damaged proteins and organelles [131]. The mitochondrial clearance process is provided by mitochondrial quality control (MQC) complexes, including AAA proteases, the ubiquitin–proteasome system, mitochondrial-derived vesicles, and mitophagy [131, 132]. A dual interaction between Parkin and PTEN-induced kinase 1 (PINK1) plays the primary role in activating MQC.

MQC activation disorders increase ROS levels and decrease ATP levels [132]. Translocase of the outer membrane 20 (TOM20) is the main protein involved in the degradation of PINK1, which may further induce mitophagy signals [133]. In the situation of mitochondrial complex insufficiency, proteotoxicity, or membrane depolarization impairment, there is PINK1 aggregation, which causes homodimerization and subsequent autophosphorylation promoting activation of kinase—all of which leads to the bonding of Parkin and ubiquitin. This complex is phosphorylated by PINK1 at Ser65, promoting E3 ligase activity [133, 134]. As a result, more Parkin is connected with ubiquitin conglomerates resulting in a higher rate of mitophagy [133, 135]. The latest studies focus on the autophagy process in neurodegenerative disease, especially in PD, where autophagy is impaired, leading to LB accumulation which cannot be removed by proteasomes [136, 137]. Additionally, the increased ROS level distorts the function of proteasomes and consequently causes the collection of an increased amount of damaged protein in the cells [138]. In dopaminergic neurons, there is a tendency toward higher levels of ROS, resulting in more sensitivity to various stress factors than other neurons.

DA may be metabolized by monoamineoxidase (MAO) [129]. The other pathway involves spontaneous DA oxidation in the presence of iron, generating 6-hydroxydopamine (6-OHDA) quinone. In the presence of oxygen, 6-OHDA is subsequently transformed into a reactive electrophilic molecule, p-quinone. There is also the possibility of oxidizing DA to DA-quinone (DAQ) [127, 139]. DAQ is described as a molecule that affects the cell’s ability to sequester ROS by reacting with Cys-106 of the neuroprotective protein DJ-1 [140, 141]. In dopaminergic neuronal cells, DJ-1 is mostly located in the cytoplasm. During a higher exposure to ROS, it is oxidized to cysteine-sulfinic (Cys-SO2H) and then translocated to the nucleus and the mitochondria [142]. However, DJ-1 has a lower efficiency in removing ROS than other enzymes, which allows us to assume that it may be an oxidative stress sensor by modulating signaling pathways and the expression of specific genes [143]. It has been indicated that the high level of DJ-1 promotes GSH synthesis and, in this way, protects DA neurons from the negative effects of H_2_O_2_ [142]_._

As a result, the change level of ROS causes the binding of DJ-1 to mitochondrial complex I and change the activity of NADH: ubiquinone oxidoreductase and, ultimately, the entire respiratory chain [144]. The disturbances in handling iron, especially in DA neurons, may result in the greatest ROS generation. The most recent studies indicate a correlation between metal ions and α-synuclein, resulting in decreased GSH levels and increased lipid peroxidation, which favors the higher production of H_2_O_2_ and hydroxyl radicals [145, 146]. All these abnormalities cause decreased ATP levels and nucleic acid damage. The main product of hydroxyl radicals is 8-oxo2dG [147]. In PD patients, the level of 8-oxo2dG compared to controls was increased in cerebrospinal fluid, serum, and urine [16]. In patients treated with L-dopa during therapy, the levels of 8-oxo2dG and corresponding DNA alterations may fluctuate depending on its duration [148].

## Epilepsy and Energy Damage

Epilepsy is one of the most common neurological diseases in the world and affects approximately 1–1.5% of the population; according to data from the World Health Organization, more than 50 million people suffer from it [149]. The prevalence of epilepsy is highest in the age group up to 10 years, especially in the first months of life and after 65 years. It is also more often diagnosed in males and developing countries [150]. However, recent epidemiological studies show that the number of patients with childhood epilepsy is decreasing while the incidence of senile epilepsy is increasing, especially after the age of 75 [151]. In 2017, the International League Against Epilepsy task force published a new, practical definition of epilepsy, thus formulating a slightly different perspective on its diagnosis [152]. According to the latest thinking, epilepsy is a brain disease that can be diagnosed in one of the following three clinical situations: when there are at least two unprovoked (or reflex) seizures that occur at least 24 h apart or when there is one unprovoked (or reflex) seizure and the likelihood of further recurrence is comparable to the overall risk of recurrence (at least 60%), such as after two unprovoked attacks that may occur in the next 10 years, and when an epileptic syndrome can be diagnosed [153].

Epilepsy is a group of symptoms that many factors can cause. According to the latest etiological classification, epilepsy is divided into genetically determined epilepsy (formerly called idiopathic), when the disease is clinically manifested mainly by epileptic seizures and is a direct result of a known or suspected genetic defect (or several mutations), e.g., the *SCN1A* gene [154]. Another type of epilepsy of structural/metabolic etiology (formerly symptomatic) is a genetically determined or acquired pathological condition that increases the risk of seizures and epilepsy of unknown cause (formerly called cryptogenic) resulting from an unexplained pathological state [153].

At the cellular level, epilepsy is the result of an imbalance between excitatory and inhibitory mechanisms, leading to paroxysmal excitation of cortical neurons. There is no one generally accepted theory of this phenomenon, i.e., epileptogenesis. This term is understood as a process that, after the action of a damaging stimulus, leads to cellular and molecular changes in the brain, resulting in the appearance of spontaneously recurring seizures. Seizures may result from structural or functional brain damage or changes in cellular metabolic pathways [155].

It is believed that metabolic and energy disorders play a crucial role in the pathogenesis of epilepsy (Fig. 2). Excessive neuronal discharges may result from the delivery of an altered energy level. In contrast, energy depletion during a seizure is an endogenous mechanism for ending a seizure. In the control of neural energy homeostasis, astrocytes play an important role via a neurometabolic linkage. Moreover, astrocyte dysfunction in epilepsy changes fundamental metabolic mechanisms. Among the disturbed metabolism associated with the astrocytes, there is, e.g., glutamate turnover, which in these central cells may directly contribute to neuronal hyperactivity. A consequence of astrocyte dysfunction is also a disturbance in the movement of energy metabolites and a disorder in removing ions (potassium) and neurotransmitters (glutamate). Astrocyte dysfunction also increases the metabolism of adenosine, the metabolic degradation product of ATP responsible for inhibiting energy-intensive processes and glucose consumption. The phenomenon of astroglial energy homeostasis in controlling neuronal excitability, related to the prevention of glucose utilization, may serve as a target for the pharmacotherapy of epilepsy [25, 156–158].

An essential role in the development of epilepsy attacks is played by disorders of neurotransmitters, especially GABA and glutamate, classified as primary messengers, which play a crucial role in the processes of stimulating and inhibiting neurons [159]. GABA is produced by the decarboxylation of glutamic acid and functions as an inhibitory neurotransmitter in the brain. Glutamate, in turn, responds to stimulation, and it is estimated that it is released in more than half of the synapses of the central nervous system [160]. The hyperactivity of the neuron is believed to result from the imbalance between glutamate stimulation and GABA inhibition [161, 162].

The reasons for the initiation and progression of epilepsy may include an increase in extracellular glutamate concentration, structural changes in the receptors for this substance (e.g., lack of the N-methyl-D-aspartic acid, NMDA NR1 subunit), impaired transport through the membrane (e.g., a reduction in the number of GLAST transporters, glutamate aspartate transporter, and GLT-1, glutamate transporter), and autoimmune mechanisms. Recent studies have shown that the concentration of pro-inflammatory cytokines—interleukins (IL) IL-1β, IL-6, and tumor necrosis factor alpha (TNF-α)—significantly increases in brain regions where epileptogenesis and signal spreading occur [163–165].

The treatment strategy for epilepsy patients is based on the long-term administration of antiepileptic drugs (AEDs) [166]. Many studies have shown that AEDs, depending on their type, can modulate the process of apoptosis and increase the concentration of Hcy. A study by Sniezawska et al. [167] showed that patients with epilepsy treated with AEDs changed not only their level of Hcy but also another risk factor for vascular diseases, ADMA, with the participation of genetic variants of genes regulating the level of Hcy, such as *MTHFR*, *MTR*, and *MTHFD1*. It has also been shown that pharmacotherapy of AEDs in patients with epilepsy increases Hcy concentration, especially in patients treated with polytherapy and long-term treatment. In addition, it has been shown that people with the *MTHFR* CT (C677T) and *MTHFD1* GG (G1958A) genotypes seem more susceptible to increased Hcy levels during AED treatment. Moreover, genetic conditions may lead to a disturbance of the ratio between Hcy and Met, arginine and ADMA, and impaired Hcy control over ADMA levels.

On the other hand, Łagan-Jędrzejczyk et al. [168] showed that in epilepsy patients with hyperhomocysteinemia treated with AEDs, the level of factors responsible for the introduction of cells to apoptosis (the ratio of Bax:Bcl-2 proteins) and cells in apoptosis increased, as measured by an increase in the level of cells with active caspase-3 using flow cytometry.

A study by Loi et al. [169] also indicated changes in apoptotic proteins in epilepsy. It showed that cells deficient in cyclin-dependent kinase-like 5 (CDKL5) increased the proapoptotic Bax protein and biomarkers associated with DNA damage (γH2AX, RAD50, and PARP1). Moreover, CDKL5-deficient cells were hypersensitive to stress associated with DNA damage, accumulated more DNA damage foci, and were more susceptible to cell death than the controls [169].

The limitations of the review are as follows:

- Common neurological diseases are only described in the review.
- Neurological diseases with neurodegenerative changes were selected.
- Diseases in which ROS generation is involved in the pathogenesis have been selected.
- Selected biomarkers of energy disorders are described.
- Non-selective biomarkers present in various pathologies of the central nervous system have been described.

## Conclusion

Although degenerative changes in neurological diseases such as stroke, AD, PD, and epilepsy have different mechanisms of central damage, affect other areas of the brain, and develop different clinical features in patients, they generally lead to an increase in oxidative stress and energy disturbances and the appearance of altered biomarker levels. The levels of factors related to energy disorders are of particular interest, including Hcy, DNA damage in the form of 8-oxo2dG, genetic variants, and antioxidants such as GSH (Table 1). Specific biomarkers are currently sought for neurological diseases related to disease manifestation, progression, and responses to available drug pharmacotherapy.

We believe that by finding specific early markers of metabolic and energy disorders, the diagnosis and treatment of neurological diseases could improve in the near future.

## Data Availability

Not applicable.

## Abbreviations

Aβ: β-Amyloid
AD: Alzheimer’s disease
ADMA: Asymmetric dimethylarginine
AEDs: Antiepileptic drugs
AGE: Glycation products
ATP: Adenosine triphosphate
C: Cytosine
CBS: Cystathionine β-synthase
CDKL-5: Cyclin-dependent kinase-like 5
CNS: Central nervous system
CO: Cytochrome c oxidase
Cys: Cysteine
Cys-SO2H: Cysteine-sulfinic
DA: Dopamine
DAQ: DA-quinone
DJ-1: Protein deglycase 1
DNA: Deoxyribonucleic acid
eNOS: Endothelial isoform of nitric oxide synthase
ET-1: Endothelin 1
GABA: Gamma-aminobutyric acid
GLAST: Glutamate aspartate transporter
GLT-1: Glutamate transporter
GSH: Glutathione
Hcy: Homocysteine
HDAC: Histone deacetylases
IL-1β: Interleukin-1β
IL-6: Interleukin-IL-6
LAA: Large-artery atherosclerosis
LB: Lewy body
L-CAR: Levocarnitine
L-dopa: Levodopa
MAO: Monoamineoxidase
MAT: Methionine adenosyltransferase
MDA: Malondialdehyde
Met: Methionine
Met/Syn: Metabolic syndrome
MQC: Mitochondrial quality control
mtDNA: Mitochondrial DNA
mtGSH: GSH in mitochondria
MMSE: Mini–Mental State Examination
MS: Methionine synthase
MTHFR: Methylenetetrahydrofolate reductase
NADH: Nicotinamide adenine dinucleotide
NMDA: N-Methyl-D-aspartic acid
NO: Nitric oxide
3-NP: 3-Nitropropionic acid
6-OHDA: 6-Hydroxydopamine
8-oxo2dG: 8-Oxo-2-deoxyguanine
OXPHOS: Oxidative phosphorylation subunits
PD: Parkinson’s disease
PGI2: Prostaglandin G2
PINK1: PTEN-induced kinase 1
QUIN: Quinolinic acid (2,3-pyridine dicarboxylic acid)
ROS: Reactive oxygen species
SAH: S-Adenosine homocysteine
SAHH: S-Adenosine homocysteine hydrolase
SAM: S-Adenosine methionine
SCFA: Short-chain fatty acids
SIRT1: Sirtuin 1
SOD: Superoxide dismutase
SVO: Small-vessel occlusion
T: Thymine
TLR-4: Toll-like receptor 4
TNF-α: Tumor necrosis factor alpha
TOM20: Translocase of the outer membrane 20
TXA2: Thromboxane A2
VitB6: Vitamin B6
VitB12: Vitamin B12
